# Supplementary material for: Epidemiological waves - Types, drivers and modulators in the COVID-19 pandemic
Source: Heliyon. 2023 May 3;9(5):e16015. doi: 10.1016/j.heliyon.2023.e16015 (PMC10154246; doi:10.1016/j.heliyon.2023.e16015)
Supplement: Multimedia component 1 [file mmc1.pdf]

Supplementary Material

Epidemiological waves - types, drivers and modulators in the COVID-19 pandemic

John Harvey[1,†], Bryan Chan[2,†], Tarun Srivastava[3,†], Alexander E. Zarebski[4], Paweł Dłotko[5], Piotr Błaszczyk[6], Rachel H. Parkinson[4], Lisa J. White[7], Ricardo Aguas[8], Adam Mahdi[9]

[1] *Department of Mathematics, Swansea University, Swansea, UK.* [2] *Department of Economics, London School of Economics and Political Science, London, UK.* [3] *Department of Engineering Science, University of Oxford, Oxford, UK.* [4] *Department of Zoology, University of Oxford, Oxford, UK.* [5] *Dioscuri Centre in Topological Data Analysis, Mathematical Institute, Polish Academy of Sciences, Warsaw, Poland.* [6] *Faculty of Computer Science, Electronics and Telecommunications, AGH University of Science and Technology, Krakow, Poland.* [7] *Li Ka Shing Centre for Health Information and Discovery, Big Data Institute, University of Oxford, Oxford, UK.* [8] *Nuffield Department of Medicine, Mahidol-Oxford Tropical Medicine Research Unit, University of Oxford, Oxford, UK.* [9] *Institute of Biomedical Engineering, Department of Engineering Science, University of Oxford, Oxford, UK.*

† JH, BC and TS contributed equally to the authorship of this manuscript.

1 Algorithm

1.1 Prominence

Prominence can be defined using mathematical notation as follows. Consider a time series given as a function f. Suppose that a local maximum occurs at the time t_0 with value $f(t_0)$. Let t_+ be the first time after t_0 when $f(t_+) \geq f(t_0)$ without f being constant on the period from t_0 to t_+. Let t_- be the latest time before t_0 when $f(t_-) \geq f(_0)$ without f being constant on the period from t_- to t_0. The parent peak of the peak at t_0 lies either before t_- or after t_+. Now let V_- be the minimum value achieved between t_- and t_0, and let V_+ be the minimum value achieved between t_0 and t_+. These are the values at which the island around t_0 merges with the islands containing t_- and t_+ respectively, one of which contains the parent peak. Note that they will have values higher than $f(t_0)$ The 'prominence' of the peak at t_0 is the lesser of the two values $(f(t_0) - V_-)$ and $(f(t_0) - V+)$. Note that precisely at this value, the peak at t_0 will merge with a more prominent peak.

We may also consider the prominence of a trough. This can be calculated simply by multiplying all values of the time series by -1. Visually, this corresponds to the water level rising and studying the merging of lakes, rather than of islands.

1.2 Reduction to minima and maxima

The algorithm does not alter the time series; rather it processes the list of local minima and maxima present in the time series. The minima and maxima are equivalent to troughs and peaks of 'wave candidates'. This list is pruned, which is equivalent to merging of the wave candidates, until each wave candidate is sufficiently significant, substantial and sustained to be termed a 'wave'. Since both peaks and troughs are important in characterising a wave, the algorithm operates in as symmetric as possible a manner with respect to minima and maxima. The focus is on identifying the timing of peaks and troughs.

From the preprocessed time series, all local minima and maxima are identified and their prominence is calculated. This procedure can be carried out using the algorithm implemented in Python as `scipy.signal.find_peaks` [1]. This returns a list of peaks in a time series along with their prominence. This allows us to create a list of quadruples, each of which is of the form `Q = (index, value, type, prominence)`. This list will be the object processed by the algorithm. The variable `index` stores the date, `value` stores the value of the time series at that date, `type` records whether the time series has a minimum or maximum at that date, and `prominence` is the prominence of that minimum or maximum. If `Q` is a quadruple, we will refer to the quadruple immediately before `Q` as `Q-` and the quadruple immediately after `Q` as `Q+`. Note that both `Q-` and `Q+` have the opposite type to `Q`.

This initial list of maxima and minima corresponds to wave-like features, many of which will be of no practical relevance owing to the large amounts of noise in the time series. Four different sub-algorithms will prune this list until only features of sufficient duration and prominence to be described as waves remain. The first two, Sub-algorithms A and B, remove features of low duration, while Sub-algorithms C and D remove features of low prominence. When, during pruning of the list, the variable `prominence` must be updated, this can be done by applying the `find_peaks` algorithm to the list of quadruples; it is not necessary to apply it to the entire time series again. A visual depiction of how this pruning process operates, step by step, is given in Figure 1.

1.3 Sub-algorithm A: Removing short waves

Description: Operates on the list of quadruples to ensure that all waves have duration at least `Tsep`. The wave duration is understood as the period between two consecutive minima or, symmetrically, between two consecutive maxima. Waves of short duration are absorbed by their neighbours until no short waves remain. Figure 1 shows how this first step removes most of the local maxima and minima.

Procedure: Proceed through the list in order of increasing `prominence`. For each quadruple `Q` in the list, consider the difference in `index` between `Q+` and `Q-`; this is the `duration` of the wave centred on `Q`. If `duration < Tsep`, delete `Q` from the list. Either `Q-` or `Q+` must also be deleted. If `Q-` and `Q+` have `type = 'maximum'`, delete the quadruple with lesser `value`. If they have `type = 'minimum'`, delete the quadruple with greater `value`. Update the `prominence` for the remaining quadruples on the list and repeat the procedure.

Outcome: After application of this Sub-Algorithm, every second entry in the list is separated by at least `Tsep`. Each entry corresponds to a peak or trough of a potential wave.

1.4 Sub-Algorithm B: Removing other short features

Description: Operates on the list of quadruples to make adjustments if a minimum and maximum occur with a difference in `index` of < `Tcen/2`, where `Tcen` $\leq$ `Tsep`. There are many possible ways for this to arise in a time series. A sudden spike in the time series superimposed on a general downward trend will generate a minimum and a swiftly following maximum, but no second minimum. A sudden drop in cases might be seen as a maximum swiftly followed by a minimum. In order to determine whether these features should be retained, this Sub-Algorithm 'censors' the data for a period of duration `Tcen`. If, after censoring this data, the fall or rise is still visible, then it will be retained. Figure 1 shows several features of this type, sometimes large in magnitude, all of which are only transient.

Procedure: Identify all quadruples `Q` so that the difference in `index` between `Q` and `Q+` is `< Tcen/2`. Let `V` and `V+` be the `value` at `Q` and `Q+`. Operate on these quadruples in order of increasing difference in `abs(V - V+)`. Calculate `t1 = (index(Q) + index(Q+) - Tcen)/2` and `t2 = (index(Q) + index(Q+) +Tcen)/2`. These are the endpoints of a window of duration `Tcen` centred over the period between `Q` and `Q+`. Sub-Algorithm A guarantees that the period from `t1` to `t2` does not contain any other quadruples. Let `y1` and `y2` be the values of the time series at times `t1` and `t2`. Compare the sign of `(V - V+)` to the sign of `(y1 - y2)`. If they disagree, delete `Q` and `Q+` from the list. If they agree, retain `Q` and `Q+`. Update the `prominence` for the remaining quadruples on the list and repeat the procedure.

Outcome: After application of this Sub-Algorithm, every second entry in the list is separated by at least `Tsep`. If two consecutive entries are separated by only `Tcen/2`, it is guaranteed that the corresponding increase or decrease has duration at least `Tcen`. Each entry corresponds to a peak or trough of a potential wave.

1.5 Sub-Algorithm C: Remove features with low prominence

Description: Operates on the list of quadruples to remove any pair of consecutive quadruples with `prominence` less than `Pabs`. Figure 1 shows how this removes a small increase in cases between two large waves.

Operation: Remove all quadruples with `prominence < Pabs`. Since the least prominent maximum is always a neighbour of the least prominent minimum, it is not relevant what order this is carried out in, and there is no need to make any recalculations.

Outcome: After application of this Sub-Algorithm, every second entry in the list is separated by at least `Tsep`. If two consecutive entries are separated by only `Tcen/2`, it is guaranteed that the corresponding increase or decrease has duration at least `Tcen`. Each entry corresponds to a peak or trough of a potential wave which has prominence at least `Pabs`.

1.6 Sub-Algorithm D: Remove features with small relative prominence

Description: Operates on the list of quadruples to remove those satisfying both `type = 'maximum'` and `prominence < Prel * value`. Note that for `value > Pabs/Prel`, peaks filtered out by Sub-Algorithm C would also be filtered out by this Sub-Algorithm. Therefore, Sub-Algorithm C is in fact only relevant when time series values are low. This Sub-Algorithm is the only portion of the algorithm which distinguishes between maxima and minima. This Sub-Algorithm has no effect in Figure 1, but good examples can be seen in the UK (Main paper, Figure 4) and the US (Main paper, Figure 5).

Operation: Proceed through the list in order of increasing `prominence`. If a quadruple `Q` has `type = 'maximum'` and `prominence < Prel * value`, then delete it. Delete also one of the two neighboring minima `Q-` or `Q+`, whichever has greater value.

Outcome: After application of this Sub-Algorithm, every second entry in the list is separated by at least `Tsep`. If two consecutive entries are separated by only `Tcen/2`, it is guaranteed that the corresponding increase or decrease has duration at least `Tcen`. Each entry corresponds to a peak or trough of a potential wave which has prominence exceeding both `Pabs` and (`Prel * value` at peak).

1.7 Testing

As a 'sanity check', an artificial time series of infections was generated from a SIR model with a period of 1,000 days and replicated 4 times. Subsequently, a substantial amount of a random uniform noise has been added to the time series. Figure 2 shows how the algorithm proposed in this paper correctly recognizes the N waves as expected. In this test `Pabs` was set to slightly exceed the maximum noise level and `Tsep` was set at 90 days to better match the dynamics of the model.

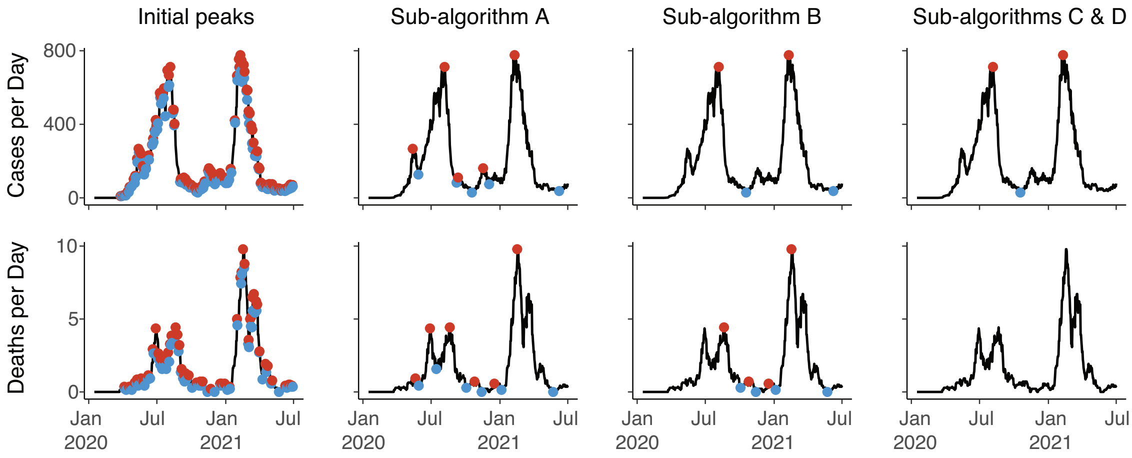

Figure 1: Illustration of how each Sub-Algorithm operates to reduce the list of identified minima and maxima in the time series of cases and deaths for Ghana. In blue, the smoothed data for each time series is shown. Solid red circles indicate which minima and maxima have survived the pruning process at each stage.

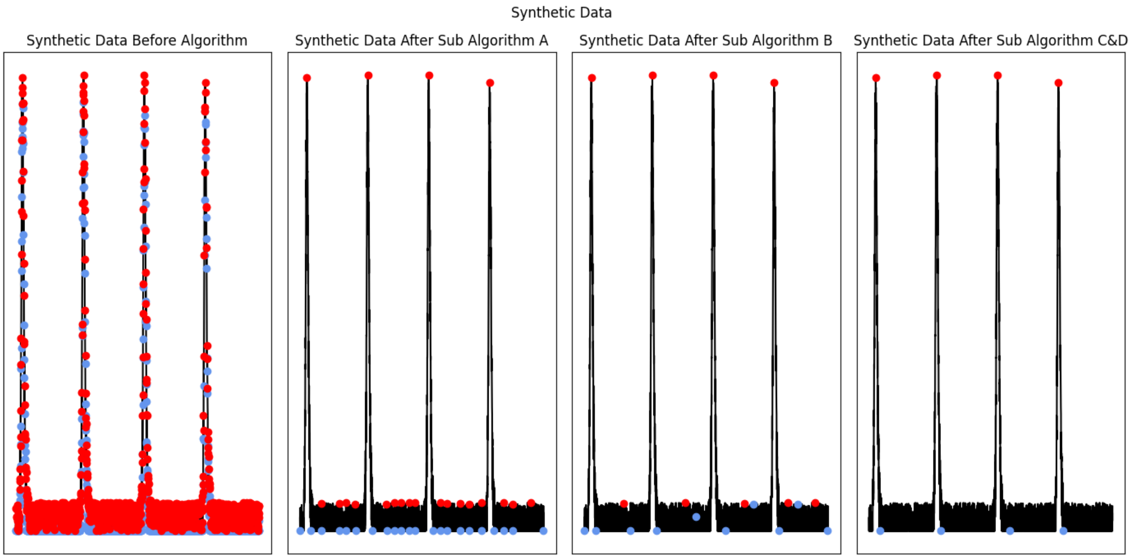

Figure 2: Illustration of how the algorithm successfully identifies four artificially generated waves.

2 Alternative thresholds

The thresholds chosen in the main body of the paper for stringency response time and for mass testing could easily have been defined differently. In Table 1 we indicate the results for some other possible choices to demonstrate the robustness of the results.

Burden metric	Response time metric	Correlation	p-value
Wave 1 Deaths	Reaching 60 on the stringency index	0.22	0.00034
Wave 2 Deaths		0.22	0.00084
All Deaths		0.25	<0.0001
Epidemic Phase		0.084	0.22
Wave 1 Deaths	Schools closing at all levels	0.21	0.00058
Wave 2 Deaths		0.25	0.00016
All Deaths		0.29	<0.0001
Epidemic Phase		0.11	0.12
Wave 1 Deaths	Workplaces closing for all but essential workers	0.12	0.082
Wave 2 Deaths		0.24	0.0011
All Deaths		0.21	0.0025
Epidemic Phase		0.16	0.037
Wave 1 Deaths	Requiring cancellation of public events	0.25	<0.0001
Wave 2 Deaths		0.23	0.00044
All Deaths		0.3	<0.0001
Epidemic Phase		0.14	0.037
Wave 1 Deaths	Restrictions on gatherings of 10 or less	0.0062	0.92
Wave 2 Deaths		0.099	0.16
All Deaths		0.053	0.41
Epidemic Phase		0.083	0.24
Wave 1 Deaths	Closing or prohibiting most citizens from using public transport	0.16	0.043
Wave 2 Deaths		0.16	0.069
All Deaths		0.22	0.0042
Epidemic Phase		0.081	0.34
Wave 1 Deaths	Requiring not leaving house with exceptions for daily exercise, grocery shopping, and 'essential' trips	0.14	0.039
Wave 2 Deaths		0.16	0.026
All Deaths		0.18	0.0048
Epidemic Phase		0.094	0.19
Wave 1 Deaths	Internal movement restrictions	0.19	0.0025
Wave 2 Deaths		0.22	0.0019
All Deaths		0.25	<0.0001
Epidemic Phase		0.11	0.12
Wave 1 Deaths	Travel ban on all regions or total border closure	0.24	0.00028
Wave 2 Deaths		0.083	0.26
All Deaths		0.24	0.00043
Epidemic Phase		0.081	0.26
Wave 1 Deaths	Availability of open public testing	0.15	0.039
Wave 2 Deaths		0.014	0.86
All Deaths		0.078	0.29
Epidemic Phase		-0.044	0.59
Wave 1 Deaths	Comprehensive contact tracing	0.18	0.0094
Wave 2 Deaths		0.044	0.55
All Deaths		0.072	0.28
Epidemic Phase		0.092	0.21
Wave 1 Deaths	1 cumulative test per 10000 population	0.32	<0.0001
Wave 2 Deaths		0.085	0.28
All Deaths		0.27	0.0002
Epidemic Phase		-0.036	0.65
Wave 1 Deaths	10 cumulative tests per 10000 population	0.22	0.0024
Wave 2 Deaths		-0.017	0.83
All Deaths		0.15	0.042
Epidemic Phase		-0.076	0.34
Wave 1 Deaths	100 cumulative tests per 10000 population	-0.0054	0.94
Wave 2 Deaths		-0.2	0.01
All Deaths		-0.13	0.082
Epidemic Phase		-0.076	0.34

Table 1: All figures are normalized to population size and response times are the days taken to achieve the measure, relative to the date that 10 cumulative deaths was reached. Correlation is Kendall's rank correlation.

References

[1] P Virtanen *et al.*. SciPy 1.0: Fundamental Algorithms for Scientific Computing in Python. *Nature Methods 17(3)* 261–272, 2020
